# *Bifidobacterium longum* subsp. *longum* dipro-O: a potential therapeutic agent for ameliorating metabolic disorders in high-fat diet-induced obesity mouse model

**DOI:** 10.3389/fendo.2025.1519058

**Published:** 2025-06-06

**Authors:** Ning Xu, Jiang Luo, Weiyang Chen, Weiwei Xiang, Yue Zhai, Wei Jiang, Junlin Wu, Yanqing Hao, Meiru Chen, Qinghua Yu

**Affiliations:** Laboratory of Microbiology, Immunology and Metabolism, Diprobio (Shanghai) Co., Ltd., Shanghai, China

**Keywords:** obesity, lipid metabolism, bifidobacterium longum, blood glucose, cholesterol, triglyceride (TG)

## Abstract

**Background:**

Excessive fat intake results in lipid metabolic disorders accompanied by inflammation and other complications. However, the effectiveness of drug interventions for metabolic disorders is not ideal, owing to their inherent limitations. Here, we introduce the probiotic *Bifidobacterium longum* subsp. *longum* dipro-O, which ameliorates metabolic disorders without any side effects.

**Method:**

C57BL/6J mice were fed a 60% kcal high-fat diet (HFD) for eight weeks to induce obesity, and then dipro-O intervention was administered for nine weeks. Blood glucose, serum cholesterol, triglyceride, and high-density lipoprotein (HDL) levels were assessed, and liver sections were processed to evaluate fat accumulation.Intestinal barrier related gene and pro-inflammatory gene in colon were detected to analyze the ability of dipro-O in intestinal homeostasis remodeling and 16S rRNA sequencing was performed to assess the changes in intestinal microbial composition.

**Result:**

After eight weeks of obesity induction, probiotic interventions were initiated and lasted for 9 weeks. Compared to the HFD-PBS group, mice in the HFD-dipro-O group gained less body weight and showed a statistically significant improvement in blood glucose control. Similarly, serum cholesterol and triglyceride levels were significantly reduced, while serum HDL was elevated, and liver sections showed that dipro-O intervention decreased fat accumulation and injury levels in the liver. Functional enrichment analysis revealed that changes in the gut microbiota inhibited bacterial invasion of epithelial cells.

**Conclusion:**

Dipro-O effectively reduced HFD-induced obesity by decreasing body weight gain, serum lipid marker levels, and liver fat accumulation. QPCR and 16S rRNA sequencing data indicated that dipro-O intervention promoted intestinal homeostasis maintenance. Taken together, these findings indicate that dipro-O has the potential to intervene in lipid disorders as an alternative to drug therapies.

## Introduction

Lipid metabolism-related diseases encompass a range of metabolic disorders, including obesity, diabetes, fatty liver disease, hyperlipidemia, atherosclerosis, cardiovascular and cerebrovascular diseases, and even certain cancers (1). Obesity, often caused by excessive caloric intake, leads to fat accumulation in tissues and organs, triggering elevated inflammatory responses and other complications (2, 3). In recent decades, the prevalence of obesity has sharply increased. Despite this, the therapeutic outcomes for obesity remain suboptimal as many treatments are associated with significant side effects (4). For instance, Orlistat, a lipase inhibitor, is limited by its mechanism of action (5). GLP-1 receptor agonists, which were initially developed for diabetes management, are now used for weight control, owing to their efficacy. However, clinical trials and cohort studies have demonstrated that the effect is dose-dependent and discontinuation often results in weight rebound (6). Furthermore, a substantial proportion of the drug-induced weight loss can be attributed to skeletal muscle reduction (7).

Recent technological advancements have facilitated the exploration of gut microbiota and its profound impact on host health. As demonstrated in previous studies, these effects begin *in utero (* 8–10), where microbial metabolites cross the placenta and influence fetal development, Following birth, these effects are further amplified as the infant encounters microbes directly for the first time. Among microbial metabolites, short-chain fatty acids (SCFAs) have been shown to play beneficial roles in immune regulation, glucose metabolism, and other physiological functions (11, 12). Beyond normal physiology, gut microbiota has also been implicated in pathological processes, including modulating emotions and alleviating depression and anxiety (13), as well as contributing to antitumor activities (14). Probiotics, defined as live microorganisms that, when administered in adequate amounts, confer health benefits to the host, have emerged as a potential therapeutic strategy owing to their ability to restore gut microbial balance and influence host biology (15). Recent studies have confirmed that specific probiotics can interfere with obesity and other related issues caused by lipid metabolism disorders. For example, a synbiotic product combining *Bifidobacterium animalis* subsp. *lactis* B420 and polysaccharides can improve gut microbiota balance, thereby exerting a positive influence on weight management (16). *Bifidobacterium breve* B3 can reduce lipid accumulation in the body by promoting fat metabolism and regulating insulin sensitivity (17). *Bifidobacterium animalis* subsp. *lactis* BPL1 (CECT8145) has been shown to reduce fat content in nematodes by 26.2% and, in clinical studies, can decrease abdominal fat deposition and enhance insulin sensitivity (18, 19). However, these strains have limitations in weight management and lipid metabolism disorder intervention; for example, their effects on improving inflammatory responses are limited, which is a common complication of obesity. Therefore, probiotics that can interfere with obesity and complications from multiple perspectives are needed.

*Bifidobacterium* is a common probiotic strain, with a long history of use and extensive research. Certain strains have been proven to possess capabilities related to lipid metabolism intervention, such as the ability to produce bile salt hydrolase and degrade cholesterol *in vitro* (20–22). In a previous study, we identified *Bifidobacterium longum* subsp. *longum* dipro-O, which exhibits remarkable bile salt hydrolase activity, cholesterol sedimentation capacity, and indole-3-lactic acid secretion *in vitro* (23), In this study, the effect of dipro-O was evaluated in a mouse model of diet-induced obesity, and the intervention efficiency was evaluated by assessing the efficiency of lipid accumulation inhibition, changes in insulin sensitivity, levels of other serum lipid metabolism-related molecules, and restoration of gut homeostasis.

## Materials and methods

### Isolation and identification of dipro-O

Dipro-O was isolated from the feces of a 12-year-old girl in China and preserved at the China General Microbiological Culture Collection Center (CGMCC) under depository number CGMCC No. 29383, dated December 25, 2023. MALDI-TOF and 16S rRNA analysis identified dipro-O as *Bifidobacterium longum* subsp. *longum*. Antibiotic susceptibility tests showed that dipro-O is sensitive to kanamycin, streptomycin, tetracycline, erythromycin, clindamycin, chloramphenicol, vancomycin, and other antibiotics (16S rRNA amplification primers are provided in **Table 1**).

**Table 1:** 16S rRNA amplification primers for *Bifidobacterium longum* subsp. *longum* dipro-O.

Name	Sequence (5’-3’)
27F	AGAGTTTGATCCTGGCTCAG
1492R	GGTTACCTTGTTACGACTT

### Activation and preparation of dipro-O for oral administration

For animal experiments, glycerol-preserved dipro-O was removed from the -80°C freezer, thawed in a 37°C water bath, and 20μL was plated on MRS+ agar (MRS broth containing 0.5 g/L cysteine). The plates were incubated anaerobically at 37°C for 48 h. After streaking with an inoculation loop and incubating for an additional 48 h, single colonies were selected and transferred to MRS+ liquid medium for expansion. For the intervention preparation, dipro-O in the exponential growth phase was collected, centrifuged, and resuspended in PBS to an OD_600_ of 3.0, corresponding to a concentration of approximately 5×10^10^ CFU/mL. The dipro-O suspension was stored anaerobically at 4°C. The reference strain *Bifidobacterium animalis* subsp. *lactis* CECT 8145 (BPL1) was administered using the same method.

### Animal experiments design

Eight-week-old male C57BL/6J mice (SPF level) were purchased from GemPharmatech Co., Ltd. (Jiangsu, China). The mice were housed in SPF IVC cages under controlled conditions of 23 ± 2°C, 50% ± 10% relative humidity, and a 12-hour light/dark cycle. The mice were divided into four groups, with six mice per group: negative control (NC) group and HFD group, including the HFD-PBS, HFD-BPL1, and HFD-dipro-O groups. The NC group was fed with a standard diet, while the other three groups were fed with a 60% kcal high-fat diet (HFD) (Research Diets, D12492i). After eight weeks of diet-induced obesity, the average body weight of the HFD group was 20% higher than that of the NC group. Subsequently, oral administration was initiated, the NC group and HFD-PBS group (positive control) were administered 200μL of PBS, while the HFD-dipro-O and HFD-BPL1 groups received 200μL of dipro-O or BPL1 suspensions, respectively. Probiotic intervention was performed for 9 weeks. Fecal samples were collected from each mouse at the end of the 17^th^ week. At the end of the experiment, all mice were euthanized for blood, liver, and colon collection. Blood samples were used for total cholesterol (TC), triglyceride (TG), high-density lipoprotein (HDL), and leptin analyses. Liver samples were collected for histological analysis and colon samples were collected for gene expression analysis. The Ethics Committee of the Animal Facility approved this study.

### OGTT test

An oral glucose tolerance test (OGTT) was conducted two days before the end of the experiment. Mice were fasted for approximately 12 h and fasting blood glucose levels were measured at baseline. After the initial blood glucose measurement, each mouse was administered a 20% glucose solution at 2 g/kg. Blood glucose levels were measured at 30, 60, 90, and 120 min after glucose administration. Blood samples were collected from the tail tip by gentle pressing to prevent hemolysis. The first drop of blood was discarded to eliminate contaminants, and the subsequent blood was directly drawn into the detection well for immediate analysis using a glucose glucometer(Roche), which is less susceptible to hemolysis interference.

### Blood triglycerides, total cholesterol, and high-density lipoprotein assays

Blood samples were collected and stored at 4°C for 3 h, then centrifuged at 7000 rpm for 10 min to separate the serum. TC, TG, and HDL levels were measured using colorimetric assay kits purchased from Elabscience Biotechnology Co., Ltd. (Wuhan, China).

### Histological evaluation

To assess HFD-induced liver damage, liver samples were fixed in paraformaldehyde (PFA) and dehydrated using a graded ethanol series. After clearing and hematoxylin-eosin staining, the tissues were embedded for pathological sectioning and the Non-Alcoholic Fatty Liver Disease Activity Score (NAS) was assessed by colleagues unrelated to the study.

### RNA extraction and intestinal barrier-related gene expression

Colonic tissues were collected for RNA extraction. The tissues were placed in TRIzol™ reagent (Invitrogen, USA), homogenized, and lysed. RNA was extracted using chloroform followed by washing with isopropanol and 70% ethanol. RNA was then reverse-transcribed into cDNA using the PrimeScript™ RT reagent Kit with gDNA Eraser (Takara, Japan). Real-time quantitative polymerase chain reaction (RT-qPCR) was performed using ChamQ Universal SYBR qPCR Master Mix (Vazyme, China) to detect the expression of *Tff3*, *Muc2*, *ZO-1*, *TNF-α*, and *β-actin* (RT-qPCR primers are provided in **Table 2**). The results were analyzed using the comparative Ct method, relative quantification was calculated using the ΔΔCt method, fold changes were expressed as 2^−ΔΔCt^, and the β-actin RNA level was chosen for normalization.

**Table 2:** RT-qPCR primers for intestinal barrier-related and pro-inflammatory genes.

Name	Sequence (5’-3’)
*Tff3-F*	ATTACGTTGGCCTGTCTCCA
*Tff3-R*	ATTACGTTGGCCTGTCTCCA
*Muc2-F*	GCTGACGAGTGGTTGGTGAATG
*Muc2-R*	GATGAGGTGGCAGACAGGAGAC
*ZO-1-F*	GTTGGTACGGTGCCCTGAAAGA
*ZO-1-R*	GCTGACAGGTAGGACAGACGAT
*TNFα-F*	GGTGCCTATGTCTCAGCCTCTT
*TNFα-R*	GCCATAGAACTGATGAGAGGGAG
*β-actin-F*	GTGACGTTGACATCCGTAAAGA
*β-actin-R*	GTAACAGTCCGCCTAGAAGCAC

### Fecal microbiome analysis

Fecal samples were collected individually at the end of the 17^th^ week and stored at -80°C. Microbiome sequencing and analysis were performed on a subset of 5 animals per group, randomly selected from the original cohort of 6 animals (24). Genomic DNA was extracted from the samples using a QIAamp Fast DNA Fecal Mini Kit (Qiagen, Germany) with the CTAB/SDS method. The bacterial 16S rRNA gene V3-V4 region was amplified using the TransGen AP221–02 Kit (TransGen, China), and the library was sequenced on an Illumina NovaSeq platform to generate 250 bp paired-end reads. Microbiome analysis was performed using DADA2 pipeline (https://benjjneb.github.io/dada2/tutorial.html). Briefly, paired-end reads were quality filtered, joined, and denoised to generate amplicon sequence variants (ASVs). Taxonomy was assigned using Naïve Bayes classifier trained on the V3–V4 region of the SILVA 138 SSU Ref NR99 dataset. Shannon, ACE, and Chao1 indices were used as indicators of alpha diversity and were calculated using the *phyloseq* package in R, while beta diversity was analyzed by principal coordinate analysis (PCoA) based on the weighted UniFrac distance computed using QIIME2. To explore the functional profiles of the gut microbiome, a Phylogenetic Investigation of Communities by Reconstruction of Unobserved States (PICRUSt) was performed based on 16S rRNA gene sequences.

### Statistical analysis

Data analysis was performed using GraphPad Prism version 10.1, and statistical significance was verified using the SPSS software. *The unpaired t-test* was used to compare the differences between two independent samples, *one-way ANOVA (followed by Tukey’s post hoc test)* was used to compare the differences between multiple groups, and the *Mann-Whitney U test* was used for alpha diversity analysis. Statistical significance was set at P < 0.05. Data are presented as mean ± standard error of the mean (SEM).

## Results

### Dipro-O effectively reduces body weight gain under HFD conditions

**Figure 1:**
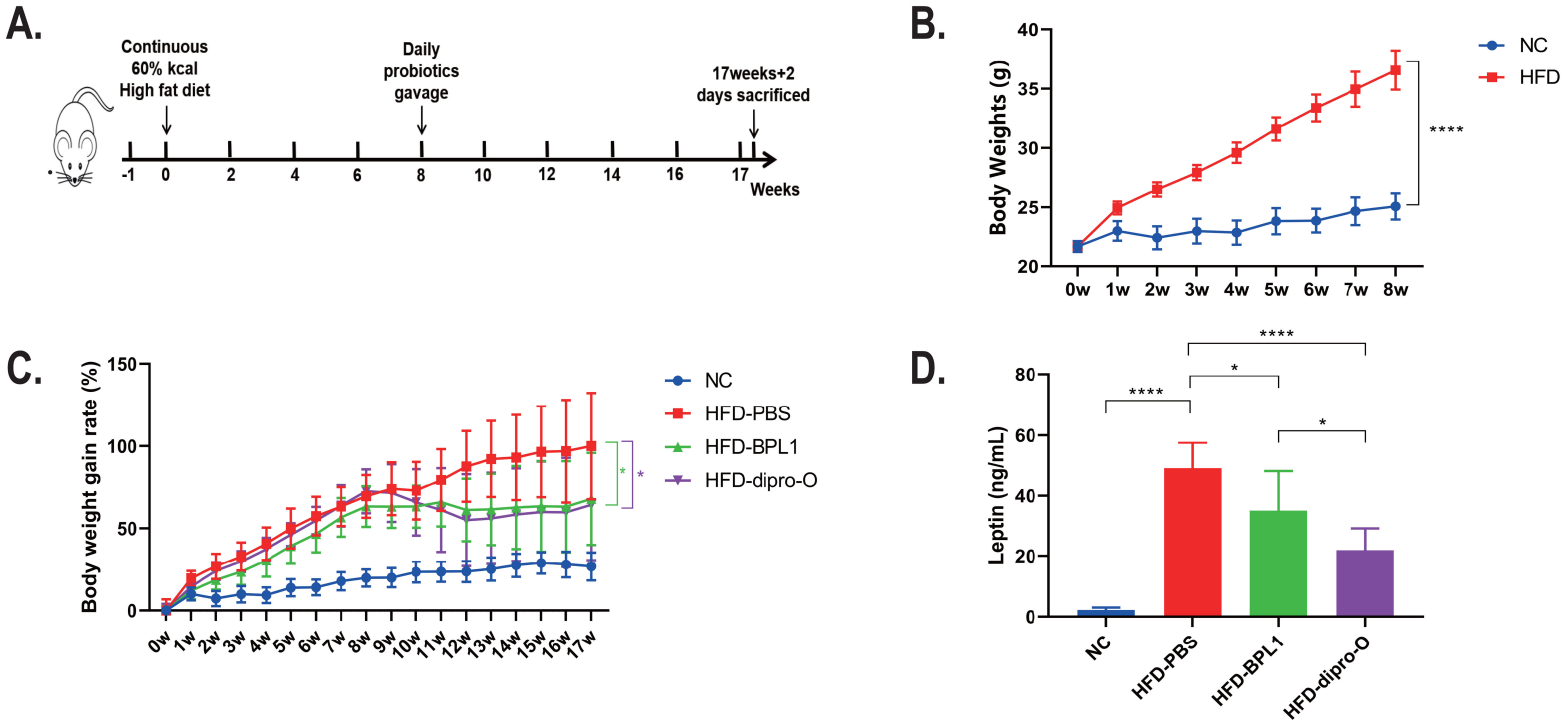
Experimental schedule and body weight changes in HFD-induced obesity model before and after dipro-O intervention. **(A)** Schedule of HFD animal model: the experiment initiated at 0 week and accomplished at 17 weeks + 2 days. **(B)** After 8 weeks HFD induction, there was a statistical difference of body weights between NC group and HFD group (NC=6, HFD group=18). **(C)** Body weight gain rates of NC, HFD-PBS, HFD-BPL1, and HFD-dipro-O groups across the experiment (n=6). **(D)** Leptin levels between NC group and HFD groups with different intervention strategies (n=6). (*P*<0.05). *P<0.05, ****P<0.0001.

The experiment lasted 17 weeks, and the study schedule is shown in **Figure 1A**. After 8 weeks of HFD induction, the body weight of the normal diet group reached 25.07 ± 1.09g, while that of the HFD group reached 36.56 ± 1.64g, which was 45.86% higher than that of the normal diet group, indicating the obesity model successful establishment and the intervention initiated (25) (**Figure 1B**). The HFD mice were divided into three groups: HFD-PBS group (treated with 200μL PBS), HFD-dipro-O group, and HFD-BPL1 group (treated with 200μL probiotic suspensions, respectively). After eight weeks of obesity induction, probiotic intervention was administered and lasted for 9 weeks. Weekly body weight measurements showed that at 17^th^ weeks, the HFD-PBS group gained 100.01% of body weight compared to the start of probiotic intervention, whereas the HFD-dipro-O group showed a significantly lower gain of 54.90%, and the HFD-BPL1 group gained 60.15%, which was also significantly lower than that in the HFD-PBS group (**Figure 1C**), and further results indicated that, compared to the HFD-PBS group, serum leptin levels in the HFD-dipro-O and HFD-BPL1 groups were significantly reduced (**Figure 1D**), and a significant difference between HFD-dipro-O and HFD-BPL1 was observed, which corresponded to the weight gain results, indicating the superior efficacy of dipro-O compared to BPL1 in obesity control under HFD conditions.

### Dipro-O improves insulin sensitivity and reduces metabolic disease-related markers

**Figure 2:**
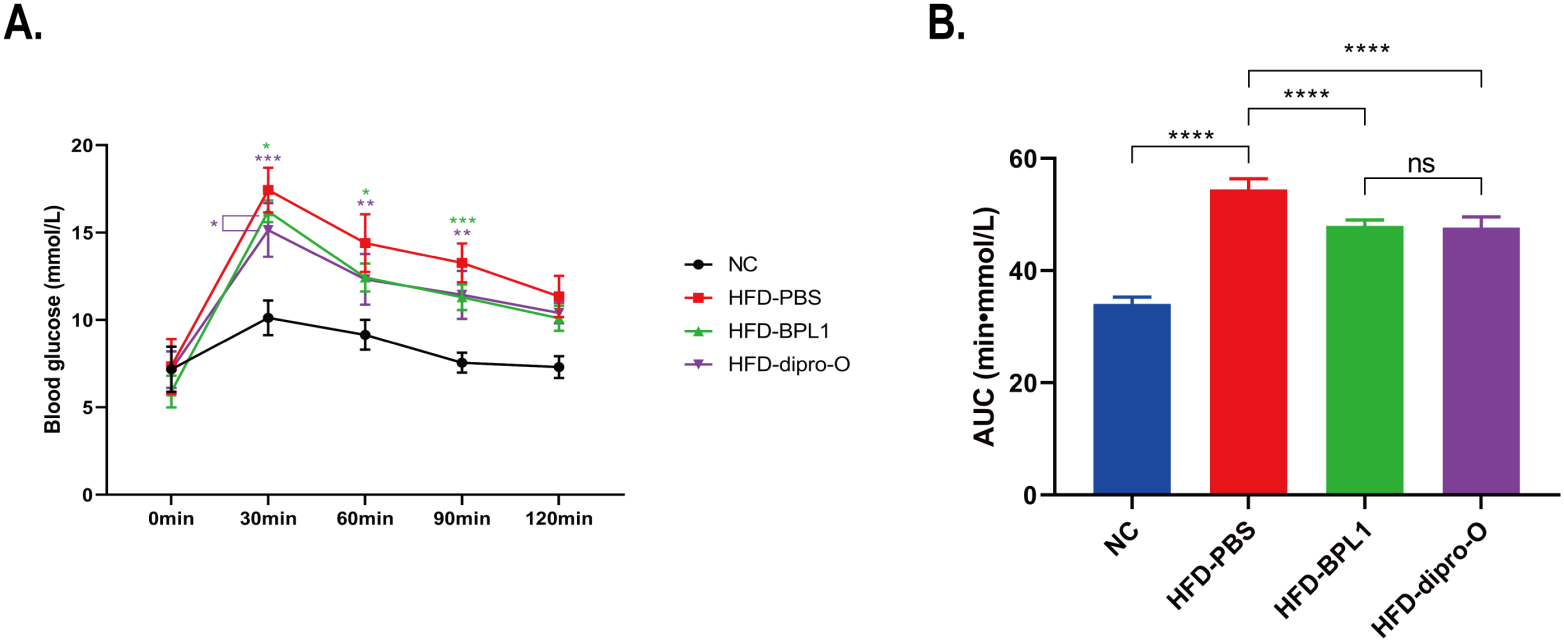
Serum glucose and lipid related molecules after dipro-O and BPL1 intervention. **(A)** The results of the OGTT experiments indicated that the HFD-dipro-O and HFD-BPL1 groups were sensitive to instant blood glucose control, and at the time point of 30 min, the HFD-dipro-O group was better than the HFD-BPL1 group. **(B)** Area Under the Curve (AUC) of OGTT. Compared to the NC group, the AUC of the HFD-PBS group was significantly elevated; however, after dipro-O and BPL1 intervention, the corresponding AUC decreased (compared with the HFD-PBS group). (n=6) (*P*<0.05). ****P<0.0001; ns, not significant.

**Figure 3:**
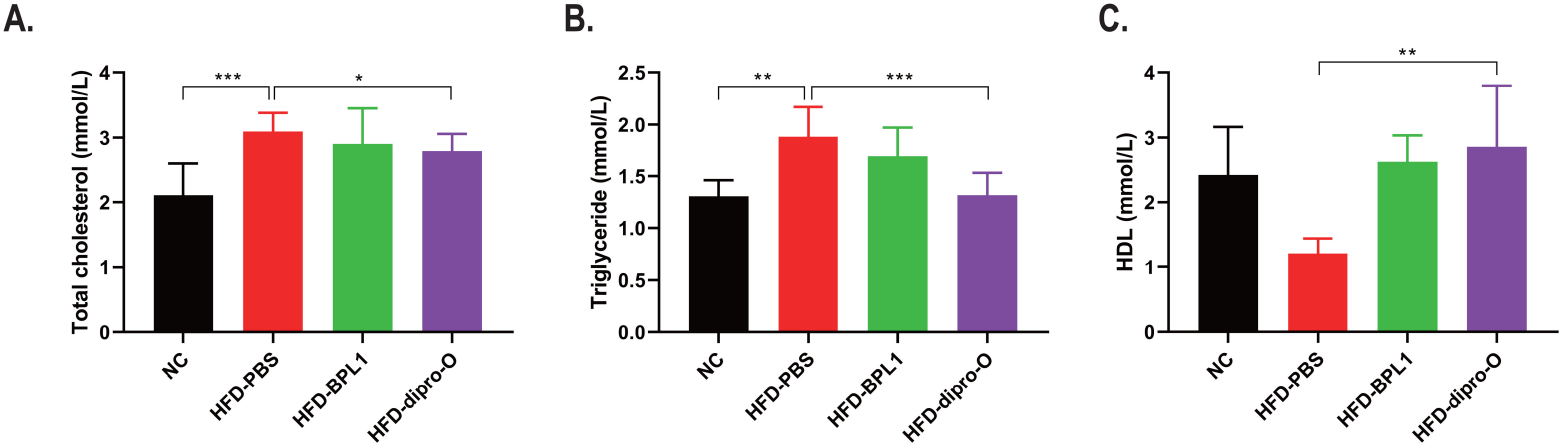
Dipro-O ameliorated HFD-induced abnormal elevation of serum lipid-related molecules. **(A, B)** After HFD induction, triglyceride and serum cholesterol levels were significantly elevated in the HFD-PBS group (compared to the NC group), while dipro-O intervention decreased triglyceride and cholesterol levels (compared to the HFD-PBS group), but there was no statistical difference between the HFD-PBS and HFD-BPL1 groups. **(C)** HDL levels were elevated after dipro-O intervention (compared to the HFD-PBS group), and no statistical difference was observed between the HFD-PBS and HFD-BPL1 groups. (n=6) (*P*<0.05). *P<0.05, **P<0.01, ***P<0.001.

At week 17, an oral glucose tolerance test (OGTT) was conducted. The data indicate that 30 min after glucose administration, the blood glucose level in the HFD-dipro-O group was lower than that in the HFD-PBS group (**Figure 2A**), consistent with area under the curve (AUC) data (**Figure 2B**). AUC data indicated that there was no significant difference between the HFD-BPL1 group and HFD-dipro-O group in general, but it is worth noting that at the 30 minutes timepoint, there was a significant statistical difference between the HFD-BPL1 and HFD-dipro-O groups, suggesting that dipro-O was more effective than BPL1 in short-term blood glucose control (**Figure 2B**). Further analysis revealed no significant differences in serum triglyceride and total cholesterol levels between the HFD-PBS and HFD-BPL1 groups. However, the HFD-dipro-O group showed significant reductions in both markers compared with the HFD-PBS group (**Figures 3A, B**). Additionally, the serum HDL level, which is known for its role in cholesterol transport to the liver, was elevated in the HFD-dipro-O group, although no significant differences were observed between the HFD-PBS and HFD-BPL1 groups (**Figure 3C**).

### Dipro-O reduces liver fat accumulation and mitigates liver injury

**Figure 4:**
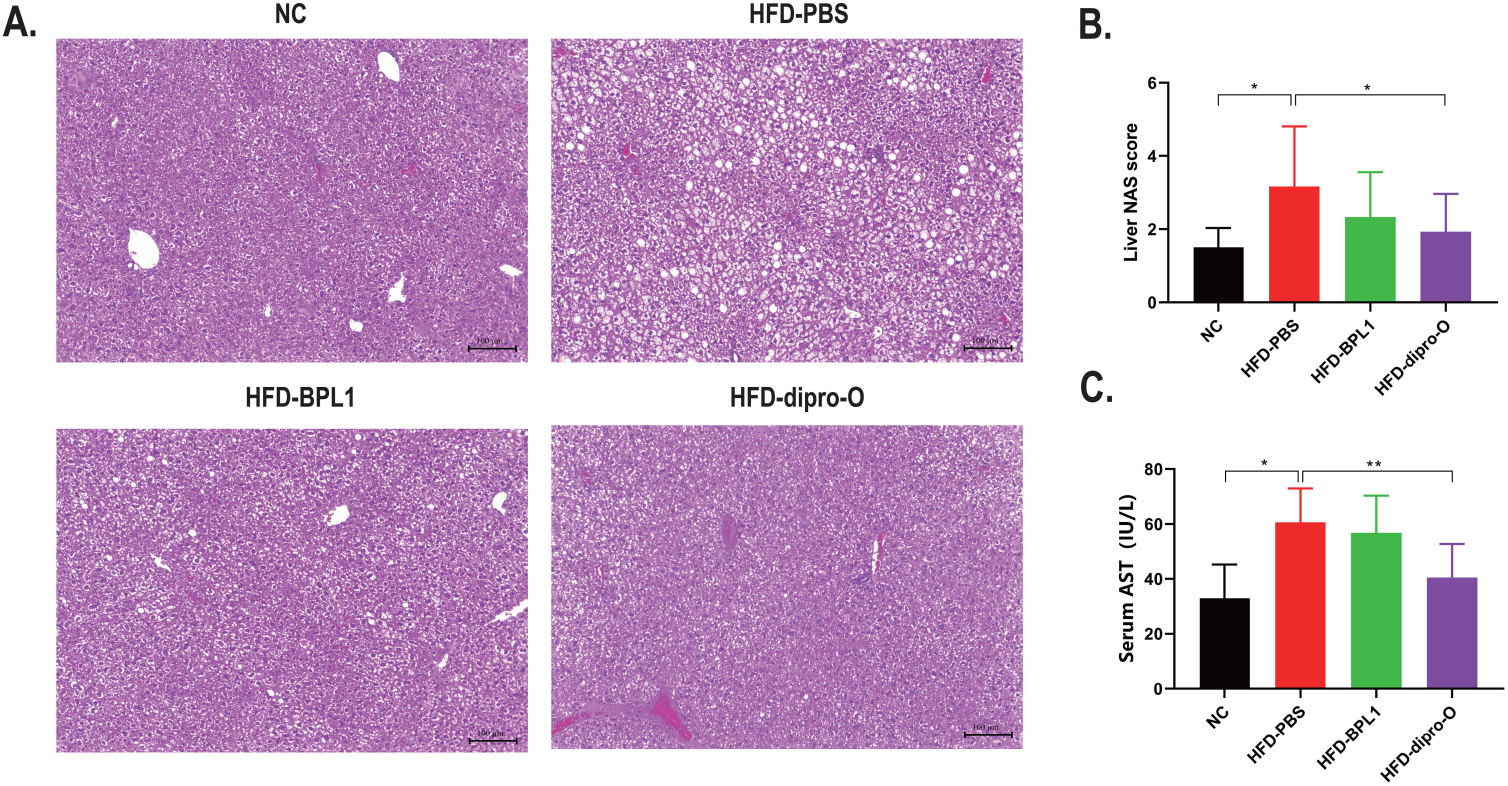
Liver injury assessment after dipro-O and BPL1 intervention. **(A)** Liver section results: HFD induced liver fat accumulation (PBS group), while dipro-O and BPL1 intervention reduced liver fat accumulation. **(B)** NAS score was assessed based on liver section to evaluate liver injury degree, and the result indicated that compared to PBS group, NAS score of dipro-O group is lower, NAS score of BPL1 group is reduced without statistic difference. **(C)** Serum *AST* level was assessed to evaluate the degree of liver injury, and the result corresponds to liver section and NAS score, dipro-O intervention ameliorated liver fat accumulation and liver injury, BPL1 is not as good as dipro-O. (n=6) (*P*<0.05). *P<0.05, **P<0.01.

Based on previous results, it was hypothesized that the reduced serum cholesterol in the HFD-dipro-O group was transported to the liver via elevated HDL. Subsequent histological analysis of the liver confirmed that compared to the HFD-PBS group, both dipro-O and BPL1 treatments inhibited liver fat accumulation and inflammatory cell infiltration (**Figure 4A**). Moreover, dipro-O treatment significantly reduced the Non-Alcoholic Fatty Liver Disease Activity Score (NAS), indicating that dipro-O does not rely on promoting fat storage to reduce serum cholesterol (**Figure 4B**). Serum aspartate aminotransferase (AST) levels showed a similar trend, with dipro-O treatment alleviating liver fat accumulation and reducing liver injury (**Figure 4C**).

### Dipro-O promotes intestinal barrier homeostasis

**Figure 5:**
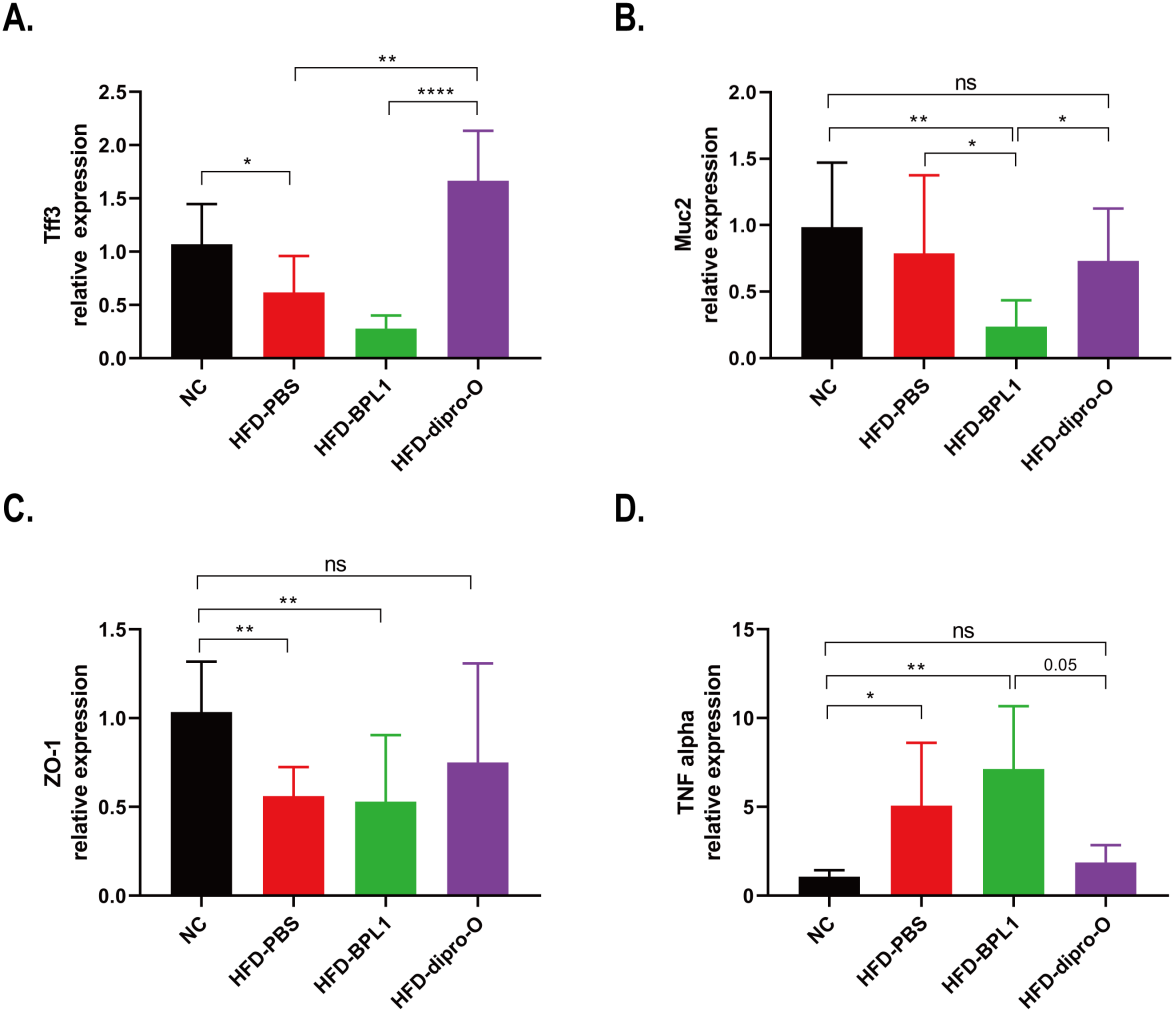
Dipro-O intervention promoting intestinal barrier integrity related gene expression while inhibiting pro-inflammatory cytokine expression in colon. **(A-C)** Colonic intestinal barrier integrity related genes *Tff3*, *Muc2*, and *ZO-1* were elevated in the dipro-O group, whereas in the PBS group, these genes were significantly decreased (compared to the NC group). **(D)** Dipro-O intervention decreased HFD induced pro-inflammatory cytokine *TNF-α* expression in colon. (n=6) (*P*<0.05). *P<0.05, **P<0.01, ****P<0.0001; ns, not significant.

The results showed that dipro-O effectively reduced body weight gain and fat accumulation in the serum and liver of HFD mice. Previous studies have demonstrated that a high-fat diet disrupts intestinal homeostasis, leading to elevated inflammatory responses and compromised gut barrier integrity (26). In this study, the expression of gut barrier-related genes and proinflammatory cytokines in the colon was assessed to evaluate the effect of dipro-O on the colon. The expression of the tight junction protein *ZO-1* was significantly elevated in the HFD-dipro-O group, but reduced in the HFD-PBS and HFD-BPL1 groups (**Figure 5A**). Similarly, the mucin protein-coding genes *Muc2* and *Tff3*, both of which are involved in the maintenance and repair of the intestinal mucosa, were upregulated in the HFD-dipro-O group (**Figures 5B, C**). Moreover, *TNF-α* expression dramatically reduced following dipro-O treatment, whereas no changes were observed in the HFD-BPL1 group (**Figure 5D**). These findings suggest that dipro-O intervention profoundly alters the intestinal microenvironment under HFD conditions, promoting a healthier gut environment for rebuilding and maintenance.

### Dipro-O treatment alters gut microbial composition and enhances biological function

**Figure 6:**
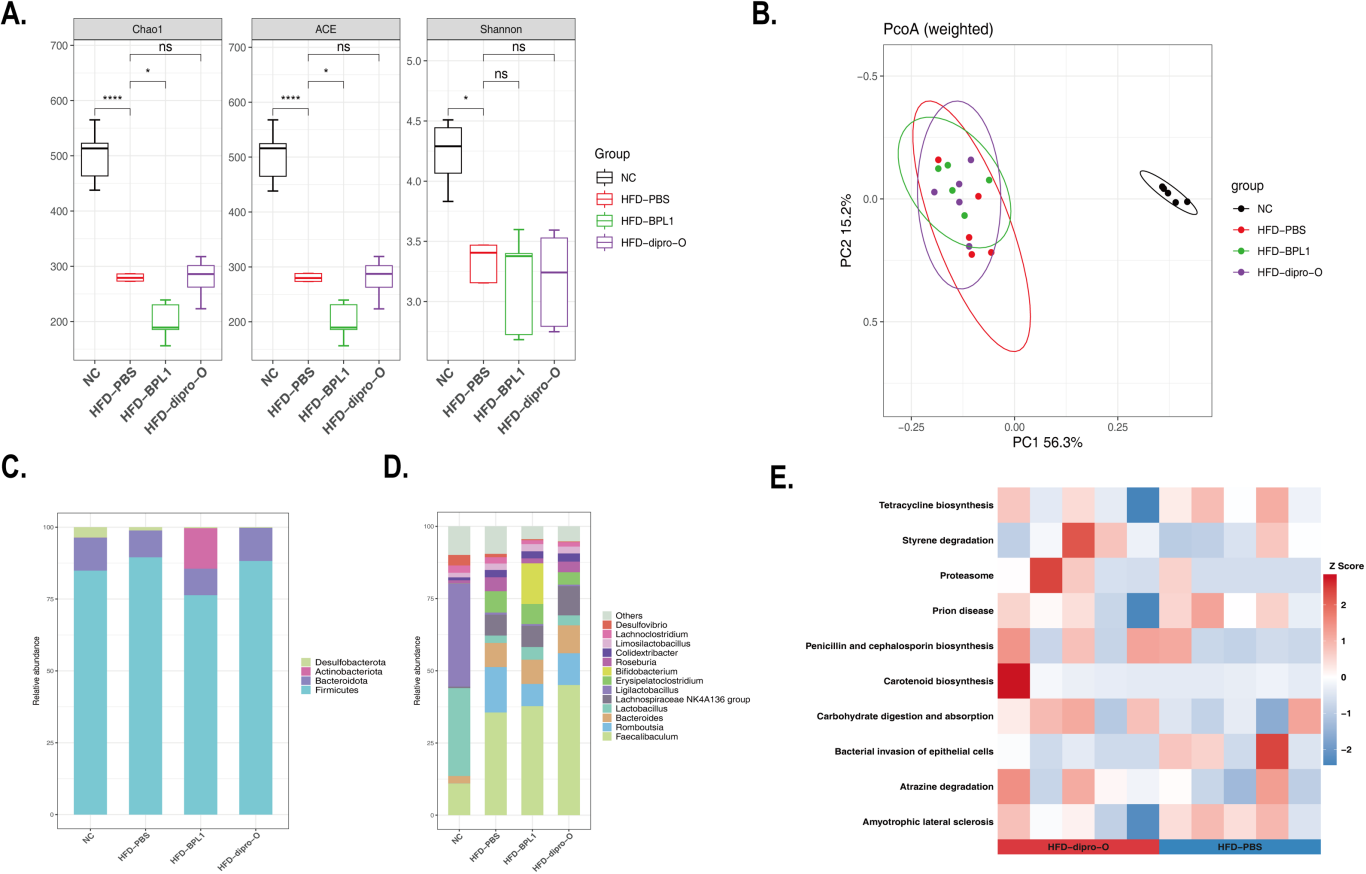
Dipro-O treatment altered microbe composition and biological function enrichment. **(A)** α-diversity of gut microbiota in NC, PBS, dipro-O and BPL1 group, data showed as Chao1, ACE, and Shannon index, respectively. **(B)** PCoA analysis was performed to explain the β-diversity alteration in the NC, PBS, dipro-O, and BPL1 groups. **(C, D)** Composition analysis at phylum and genus levels. **(E)** Functional enrichment analysis of the dipro-O. Data are presented for 5 randomly selected animals per group (n=5) (*P* < 0.05).

The α-diversity of gut microbiota in HFD mouse models treated with different interventions was assessed using the Shannon and Chao1 indices. As shown in **Figure 6A**, the diversity of gut microbiota was reduced in the HFD groups compared to the normal diet (NC) group, and neither dipro-O nor BPL1 intervention significantly altered this decreasing trend. Principal coordinate analysis (PCoA) of β-diversity revealed a clear separation between the NC and HFD groups along the first principal coordinate (PC1), indicating that a high-fat diet significantly altered the gut microbiota composition. In the HFD groups, the gut microbiota of the dipro-O- and BPL1-treated mice clustered more closely than in the HFD-PBS group (**Figure 6B**). At the phylum level, the abundance of *Desulfobacterota* decreased, while *Bacteroidota* increased in the HFD group. Notably, the abundance of *Actinobacteriota* was reduced in the BPL1 group (**Figure 6C**). At the genus level, HFD resulted in a reduction in *Lactobacillus*, while the abundance of *Faecalibaculum*, *Romboutsia*, *Bacteroides*, and *Lachnospiraceae* was elevated. Additionally, increased *Bifidobacterium* levels were observed in the HFD-BPL1 group (**Figure 6D**). Functional enrichment analysis linked these microbial changes to biological functions, showing that dipro-O treatment inhibited the bacterial invasion of epithelial cells and promoted carbohydrate digestion and absorption (**Figure 6E**). No significant beneficial effects were observed in the HFD-BPL1 group.

## Discussion

Excessive intake is known to be the reason of a series of fat-related diseases; however, drug treatment is not an ideal therapeutic strategy. In a previous study, a strain of *Bifidobacterium longum* subsp. *longum* dipro-O was isolated and demonstrated a sufficient capacity for lipid reduction *in vitro*. In this study, further investigation demonstrated its superior effects in promoting insulin sensitivity, preventing hypercholesterolemia, hypertriglyceridemia, fat accumulation in the liver, and body weight gain in a high-fat diet (HFD) animal model compared to the HFD-PBS-treated group, as well as the commercial probiotic *Bifidobacterium animalis* subsp. *lactis* BPL1 (CECT8145)-treated group. After eight weeks of HFD induction, the mice in the HFD group were approximately 45.86% heavier than those on a normal diet, and probiotic intervention was initiated. In future research, additional adiposity-related targets will be examined to confirm successful obesity induction, thereby strengthening the validity of the conclusions. During the intervention period, we observed that dipro-O treatment significantly inhibited fat accumulation compared to that in the HFD-PBS group, with an even better effect than that in the HFD-BPL1 group. The serum leptin level of the HFD-dipro-O group was significantly lower than those of the HFD-PBS and HFD-BPL1 groups, indicating that dipro-O possesses a superior capacity for weight control under HFD conditions. The OGTT results indicated that dipro-O intervention improved insulin sensitivity under HFD conditions and that dipro-O was superior to BPL1 in terms of short-term blood glucose control. However, in the OGTT test, although tail-tip sampling is a widely accepted method for serial blood glucose measurements in mice, the possibility of hemolysis still exists. To mitigate this, future studies could employ alternative sampling techniques (e.g., retro-orbital or jugular vein puncture) to avoid hemolysis. While our OGTT results demonstrated improved glucose tolerance in the HFD-dipro-O group, the absence of insulin level measurements or HOMA-IR analysis limited our evidence chain to fully characterize insulin sensitivity after dipro-O intervention. Future studies will incorporate these metrics in a more comprehensive assessment of metabolic health. In following study, the result of serum analysis in HFD-dipro-O group showed a reduction in cholesterol and triglyceride levels compared to HFD-PBS group, suggesting that dipro-O has the potential to regulate fat absorption and metabolism. Liver histology data confirmed this hypothesis, as dipro-O treatment reduced fat accumulation and ameliorated liver injury, which corresponded to lower serum AST levels.

Further investigations were conducted to explore how dipro-O regulates fat absorption and metabolism. A previous study indicated that a high-fat diet results in gut microbiota dysbiosis, characterized by changes in the composition of the gut microbiota and reduced microbial diversity, which in turn alters the metabolic products of gut bacteria, such as the reduction of short-chain fatty acids (SCFAs), increase in lipopolysaccharides (LPS), and compromised tight junction proteins. A compromised barrier allows for LPS crossing and microbial translocation, which then activates the immune system, further exacerbating dysbiosis and finally leading to a vicious cycle that promotes chronic inflammation and disease development. This bidirectional relationship highlights the importance of maintaining a balanced microbiota and robust intestinal barrier to prevent systemic immune activation and the onset of various inflammatory conditions (27–30). Therefore, we examined the expression of intestinal barrier integrity-related genes and found that compared to the HFD-PBS group, genes such as *Tff3* (a bioactive peptide involved in mucosal repair) and *ZO-1* (a tight junction protein) were upregulated in the HFD-dipro-O group. No significant difference was observed between the NC and HFD-dipro-O groups in *Muc2* (a mucin protein) expression, which was decreased in the HFD-BPL1 group (compared to the NC and HFD-PBS groups, respectively). These findings suggest that dipro-O intervention is conducive to maintaining intestinal barrier homeostasis, thereby preventing endotoxins in the gut from entering the systemic circulation and reducing the inflammatory response, which is consistent with the reduced colonic *TNF-α* expression after dipro-O intervention, indicating attenuated local inflammation. While *TNF-α* itself may not directly regulate fat absorption, the reduction in *TNF-α* levels likely contributes to the restoration of intestinal barrier integrity, as evidenced by the upregulation of *ZO-1* and *Muc2* expression, thereby reducing excessive fat absorption and the occurrence of secondary metabolic disturbances, including hepatic lipid accumulation and dysregulated lipid metabolism. In addition, in a previous study, dipro-O validated the capacity of Indole-3-lactic acid (ILA) production, which was demonstrated to alleviate intestinal inflammation by inhibiting macrophage aggregation and Treg cell differentiation under high-fat diet conditions, whereas Treg cells were reported to secrete *IL-10*, which may result in elevated *IL-10* expression in the colon. However, *IL-10* expression was not detected in this study. Future studies should include quantification of *IL-10* to further elucidate the immunomodulatory mechanisms underlying the protective effects of dipro-O on gut barrier integrity. Moreover, ILA have been reported to promote intestinal barrier-related gene expression to restore intestinal barrier balance and promote lipid metabolic-related gene expression in the liver, thereby reducing fat accumulation (31, 32). These findings may partially explain how dipro-O mediates fat reduction and maintenance of intestinal homeostasis. The amelioration of fat accumulation and the reduction in lipid-related molecules may enhance insulin sensitivity (33). Additionally, we collected fecal samples for 16S rRNA sequencing to determine whether dipro-O affects gut microbial composition, which may alter the gut microenvironment and, in turn, impact host fat absorption and metabolism. Our results showed that the dipro-O group exhibited the highest abundance of *Faecalibaculum* at the genus level, although this bacterium was previously reported to increase in response to a high-fat diet (34). Functional enrichment analysis revealed that changes in the gut microbiota (dipro-O intervention) inhibited bacterial invasion of epithelial cells, which is consistent with our finding that dipro-O reduced *TNF-α* expression in the colon.

Currently, most probiotics are limited to a small number of species or subspecies that are generally regarded as safe for use in food and supplements with few side effects. However, advances in science and technology have led to a deeper understanding of the relationship between the host and gut microbiota, as well as the roles that gut microbes play in both physiological and pathological processes. Probiotics are now recognized not only for their role in food but also for their potential in gut restoration and disease management. For instance, fecal microbiota transplantation (FMT) has been successfully used to treat *Clostridium difficile* infections, although the underlying mechanisms remain unclear (35). Furthermore, the impact of individual microbes on health has become clearer. For example, *Lactobacillus plantarum* metabolites derived from tryptophan have been shown to regulate epigenetic modifications in dendritic cells, enhance CD8+ T cell immunity, and inhibit tumor growth (36). Environmental factors, such as cadmium (Cd) pollution, can disrupt intestinal barrier function, potentially leading to bacteremia. Remarkably, the gut colonizer *Akkermansia muciniphila* has been shown to restore intestinal homeostasis by repairing goblet cell counts and ameliorating Cd-induced intestinal barrier damage (37). In addition, certain gut microbes exert effects beyond the digestive system. For example, metabolites produced by *Bifidobacterium longum* and *Roseburia intestinalis*, such as homovanillic acid (HVA), can cross the blood-brain barrier, enhance synaptic integrity, protect hippocampal neurons, and alleviate depressive symptoms (38).

In this study, we demonstrated that *Bifidobacterium longum* subsp. *longum* dipro-O effectively reduced fat accumulation, ameliorated serum lipid-related biomarkers, and increased insulin sensitivity in an HFD mouse model. Although we conducted preliminary investigations into the underlying mechanisms, the exact pathways through which dipro-O modulates intestinal barrier integrity-related genes remain unclear and will be the focus of future research. In future research, the main direction is to explore the specific mechanism of dipro-O intervention in obesity, and shall focus on the interaction and the effect of dipro-O intervention with other microorganisms that colonize the gut, as well as the mechanisms by which dipro-O restores the intestinal barrier and regulates lipid absorption *in vivo*. Although medications play a central role in disease therapy, their potential side effects often limit long-term use. We believe that maintaining gut homeostasis through probiotic interventions could become a mainstream approach for health management in the future.

## Data Availability

The datasets generated and/or analyzed during the current study are available from the corresponding author upon reasonable request.
